# Underdamped longitudinal soft modes in ionic crystallites—lattice and charge motions observed by ultrafast x-ray diffraction

**DOI:** 10.1063/4.0000143

**Published:** 2022-03-08

**Authors:** Isabel Gonzalez-Vallejo, Azize Koç, Klaus Reimann, Michael Woerner, Thomas Elsaesser

**Affiliations:** Max-Born-Institut für Nichtlineare Optik und Kurzzeitspektroskopie, 12489 Berlin, Germany

## Abstract

Soft modes in crystals are lattice vibrations with frequencies that decrease and eventually vanish as the temperature approaches a critical point, e.g., a structural change due to a phase transition. In ionic para- or ferroelectric materials, the frequency decrease is connected with a diverging electric susceptibility and, for infrared active modes, a strong increase in oscillator strength. The traditional picture describes soft modes as overdamped transverse optical phonons of a hybrid vibrational-electronic character. In this context, potassium dihydrogen phosphate (KH_2_PO_4_, KDP) has been studied for decades as a prototypical material with, however, inconclusive results regarding the soft modes in its para- and ferroelectric phase. There are conflicting assignments of soft-mode frequencies and damping parameters. We report the first observation of a longitudinal underdamped soft mode in paraelectric KDP. Upon impulsive femtosecond Raman excitation of coherent low-frequency phonons in the electronic ground state of KDP crystallites, transient powder diffraction patterns are recorded with femtosecond hard x-ray pulses. Electron density maps derived from the x-ray data reveal oscillatory charge relocations over interatomic distances, much larger than the sub-picometer nuclear displacements, a direct hallmark of soft-mode behavior. The strongly underdamped character of the soft mode manifests in charge oscillations persisting for more than 10 ps. The soft-mode frequency decreases from 0.55 THz at *T *=* *295 K to 0.39 THz at *T *=* *175 K. An analysis of the Raman excitation conditions in crystallites and the weak damping demonstrate a longitudinal character. Our results extend soft-mode physics well beyond the traditional picture and pave the way for an atomic-level characterization of soft modes.

## I. INTRODUCTION

Soft modes are particular optical phonons occurring at low frequencies in polar and/or ionic crystals. Soft-mode excitations display a hybrid character, which is characterized by concerted nuclear and electronic displacements, the latter having a strong impact on the macroscopic electric properties of the crystal. The soft-mode frequency approaches zero when the crystal structure becomes unstable,^1,2^ frequently connected with a divergence of the dielectric function,^3,4^ and, in the case of infrared-active optical phonons, a strong increase in optical oscillator strength. Such behavior has been observed in materials undergoing a transition between a para- and a ferroelectric phase at a critical temperature *T_C_*.

The classical core-shell model introduced by Cochran^5,6^ treats this scenario by solving the mechanical equations of motion of two coupled ions, one of them having a polarizable electron cloud. In this picture, the soft mode is a transverse optical phonon with a frequency approaching zero at *T* = *T_C_*, in parallel to the divergence of the static dielectric function. The coupled nuclear and electronic motions account for basic dielectric properties of ferroelectrics and, in particular, allow for including local-field effects according to the Clausius–Mossotti relation.^7^ It has been shown for prototypical ferroelectrics such as perovskites that the electric polarization is strongly dominated by electronic motions.^8^

Vibrational spectroscopy of soft modes gives at best indirect insight in the related relocation of electronic charge. In contrast, x-ray diffraction maps electronic charge density directly via the scattering structure factor. Recent x-ray powder diffraction experiments with femtosecond time resolution have provided transient electron density distributions in electronically excited states of polar crystals. Upon displacive excitation of coherent low-frequency lattice motions, electronic charge is periodically shifted over large interatomic distances, while the displacement of atoms from their equilibrium positions is orders of magnitude smaller.^9–11^ This behavior is in qualitative agreement with the core-shell model. The very limited information on the electronic structure of excited states, however, makes a more detailed analysis and clear identification of soft modes difficult. Thus, ultrafast diffraction experiments addressing coupled nuclear-electronic motions in the electronic ground state are requested for a more specific understanding of the complex spatiotemporal dynamics of soft modes.

**FIG. 1.**
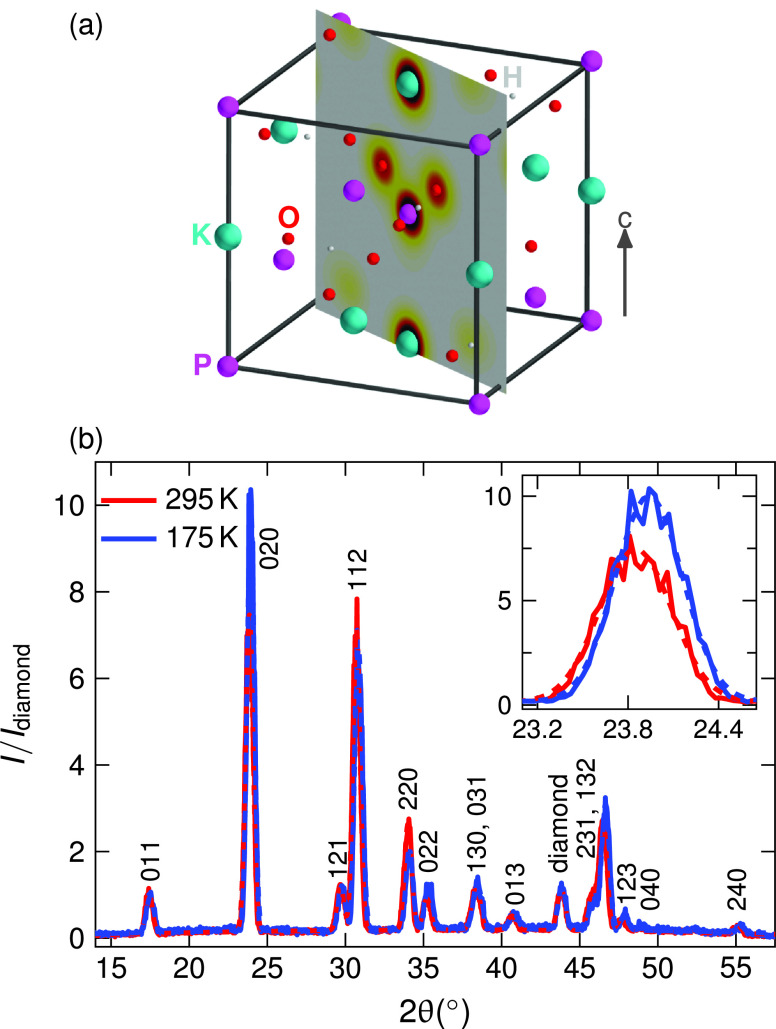
(a) Unit cell of paraelectric KDP with K, P, O, and H atoms. The transient charge-density maps shown in Fig. 6 are sectional views in the gray plane. (b) Measured stationary diffraction pattern of KDP at 295 K (red) and at 175 K (blue). Dashed lines correspond to a fit to the data. Inset: zoom on the (020) diffraction peak.

Potassium di-hydrogen phosphate (KH_2_PO_4_, KDP) has raised strong interest in this context, mainly as a prototypical material undergoing a para- to ferroelectric phase transition at $T_{C}=$ 123 K.^3,12^ In the paraelectric (PE) phase, KDP crystallizes in the tetragonal space group $I\bar{4}2d$ with four formula units per unit cell and symmetric positions of the K^+^ ions and P atoms along the *c* axis [Fig. 1(a)]. In contrast, the orthorhombic crystal structure of ferroelectric (FE) KDP (space group *Fdd*2) displays positions of K^+^ and P, which are asymmetrically shifted, resulting in a ferroelectric polarization along the *c* axis.

Early theoretical work has introduced a soft-mode model for PE KDP according to which translational motions of the K^+^ and $\text{P}{\text{O}}_{4}^{3-}$ ions along the *c* axis (*B*_2_ symmetry) couple to a collective hydrogen tunneling mode between neighboring $\text{P}{\text{O}}_{4}^{3-}$ tetrahedra.^13^ In this picture, ordering of hydrogen atoms in their double-minima potentials makes the soft-mode frequency approach zero at *T_C_*. From the experimental side, potential soft modes of KDP have been investigated for decades by stationary infrared and Raman spectroscopy,^14–21^ femtosecond impulsive Raman scattering,^22^ and dielectric spectroscopy at subterahertz frequencies in a wide temperature range.^23–26^ A number of low-frequency phonons of PE KDP show pronounced frequency down-shifts with decreasing temperature, albeit not to zero at *T* = *T_C_*, and a strong enhancement of the absorption strength in infrared active modes.

The existing spectroscopic data have mostly been analyzed with an adiabatic separation of electronic and nuclear degrees of freedom. In particular, ionic motions in crystals have been linked to the macroscopic electric polarization by introducing large Born effective charges of the ions. In this picture, the displacements of ions define the spatial range over which electrons move. This approach neglects the non-adiabatic mixed nuclear-electronic character of a soft mode, which results in vastly different length scales of nuclear and charge motions. Moreover, it makes nuclear motions susceptible to damping via electron–electron and electron-phonon interactions. There is a substantial spread of phonon frequencies obtained from the different existing measurements and broad range of damping parameters extracted from numerical fits of vibrational line shapes. While the older literature mainly claims overdamped soft-mode characteristics,^14–21^ the femtosecond Raman data of Ref. 22 suggest an underdamped response of a soft mode at a frequency of 0.6 THz (*T *=* *270 K). Overall, there is no consistent picture of the transverse soft mode of KDP. Moreover, soft modes of a longitudinal character have remained unexplored in KDP and other polar materials.

In this article, we present a study of soft-mode behavior in the electronic ground state of PE KDP crystallites, combining femtosecond x-ray powder diffraction and linear terahertz (THz) spectroscopy of low-frequency phonons. We identify a phonon of B_2_ symmetry as soft mode, which displays a frequency of 0.55 THz at *T *=* *295 K and 0.39 THz at *T *=* *175 K. In a powder sample, excitation of this mode via femtosecond impulsive Raman scattering results in underdamped coherent oscillations, which are connected with a periodic charge transfer between the potassium (K^+^) and phosphate (H_2_ $\text{P}{\text{O}}_{4}^{-}$) ions and from the P to the O atoms in the phosphate groups. The length scale of charge transfer exceeds the vibrational amplitudes by three orders of magnitude. Due to the boundary conditions set by the micrometer dimension of the crystallites, this phonon is of a longitudinal optical (LO) character. The persistence of coherent oscillations on a 10-ps timescale demonstrates its markedly underdamped character, originating from the small number of relaxation pathways existing at subterahertz phonon frequencies.

## II. EXPERIMENTAL METHODS

KDP in its PE phase has a tetragonal symmetry ( $I\bar{4}2d$) with lattice parameters a= 745.2 pm and c= 697.4 pm [Fig. 1(a)].^27^ KDP crystallites with a 99.99% purity (Alfa Aesar) were ground into a fine powder and pressed into a pellet shape. The size of the crystallites was approximately 1 *μ*m, and the thickness of the pellet was 135 ± 10 *μ*m. Since KDP is a highly hygroscopic material, the pellets were prepared under an inert Ar atmosphere and placed into a metallic holder between two thin windows of fused silica (10 *μ*m) and diamond (20 *μ*m). The diffraction experiments at *T *=* *295 K were performed under a N_2_ atmosphere.

For low temperature measurements, the sample was cooled down with a cryogenic nitrogen jet (Cryojet, Oxford Instruments). The temperature was systematically verified from the known temperature dependent lattice constants^28^ by following changes of the stationary x-ray diffraction pattern as measured with the femtosecond hard x-ray source [Fig. 1(b)].

Ultrafast optical pump-x-ray diffraction probe experiments were performed to follow charge relocation driven by lattice motions in the PE phase.^29^ The pump pulses centered at λ= 800 nm ( $E_{\text{pump}}=$ 1.55 eV), i.e., far below the bandgap of $E_{g}\approx 7$ eV, impulsively excite all Raman-active phonons in the electronic ground state, which have frequencies within the excitation bandwidth of some 12 THz. For low-frequency phonons, this mechanism generates coherent superpositions of several excited phonon states, resulting in coherent wavepacket motions along such vibrational coordinates.

The 800-nm pump pulses were generated in an amplified femtosecond Ti:sapphire laser system with a spectral bandwidth of Δλ=25 nm (FWHM), a duration of less than 50 fs, and a peak intensity of I =2 TW/cm^2^ on the powder sample. The pump spot size on the sample was ∼600 *μ*m, resulting in an incident fluence of 80 mJ/cm^2^. Femtosecond hard x-ray pulses were generated by focusing 800 nm pulses of 3.5 mJ energy on a 15 *μ*m thick copper target. The x-ray pulses at $E_{\text{xray}}=8.05$ keV (Cu K_*α*_ radiation) had a temporal width of ∼100 fs and were focused with a Montel multilayer x-ray optic (Incoatec) onto the powder sample, resulting in a spot size of 90 *μ*m (FWHM).

To ensure a homogeneous excitation and a constant temperature, the sample was continuously rotated during the experiments. The temporal delay between pump and probe pulses was set by a mechanical delay line in the pump arm of the setup. Delay times were generated in a random sequence to reduce the impact of long-term drifts on the measured pump-probe signals. The x-ray diffraction patterns were collected on a large-area detector (Pilatus, Dectris 1M). A chopper with a 25 Hz rate was used to collect diffraction patterns with and without excitation sequentially. The diffraction experiment operates close to the shot-noise limit set by the number of x-ray photons counted, as has been discussed in detail in Ref. 30.

The x-ray experiments were complemented by measurements of stationary THz spectra of pellets made of KBr and KDP crystallites. Transmission spectra at *T *=* *295 K were recorded in a frequency range up to 3 THz, using a commercial time-domain THz spectrometer (Menlo) and analyzed with the formalism described in Appendix A.

## III. RESULTS

### A. Experimental results

The stationary powder diffraction pattern of KDP in the PE phase exhibits 14 diffraction peaks up to a diffraction angle of 2θ=58°. In Fig. 1(b), the intensity integrated over the different Debye–Scherrer rings diffracted from the unexcited sample is plotted as a function of 2θ. A reduction of the sample temperature from *T *=* *295 K to 175 K results in slight angular shifts of the different peaks, as exemplified for the (020) reflection in the inset. The angular width of the peaks is determined by the experimental geometry, in particular the spot size of the x-ray beam on the sample.

**FIG. 2.**
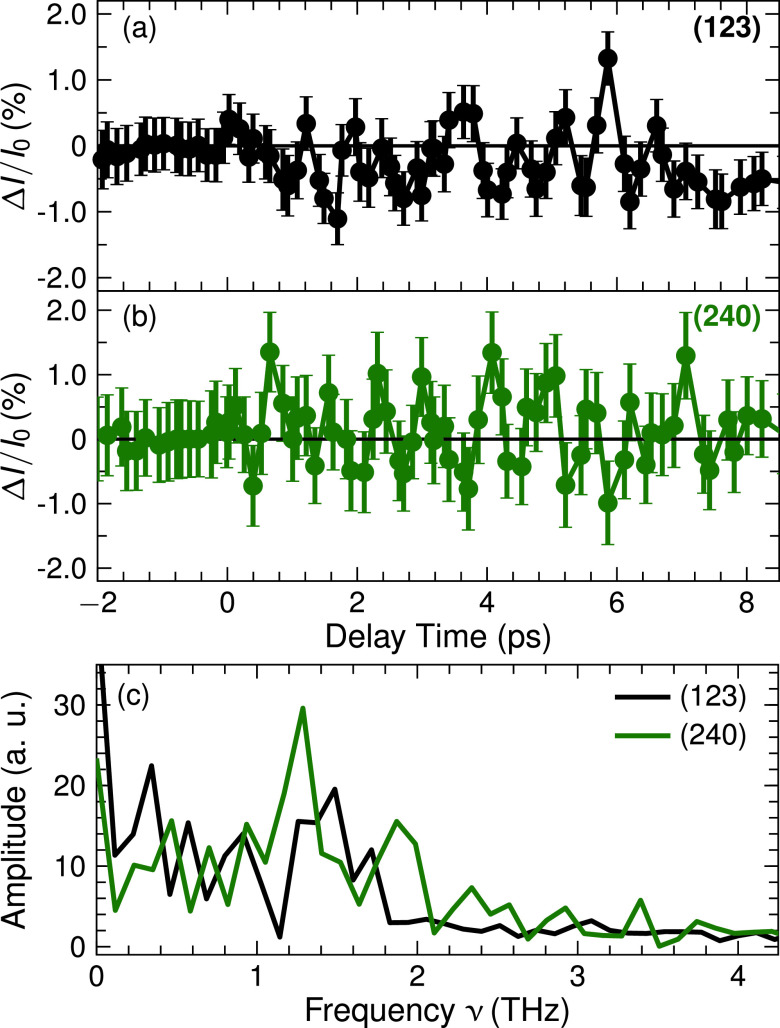
Transient intensity changes observed on the indicated powder diffraction peaks from KDP at *T *=* *295 K binned over a time interval of 200 fs (symbols). (c) FFT of the transient intensity changes in (a) and (b).

**FIG. 3.**
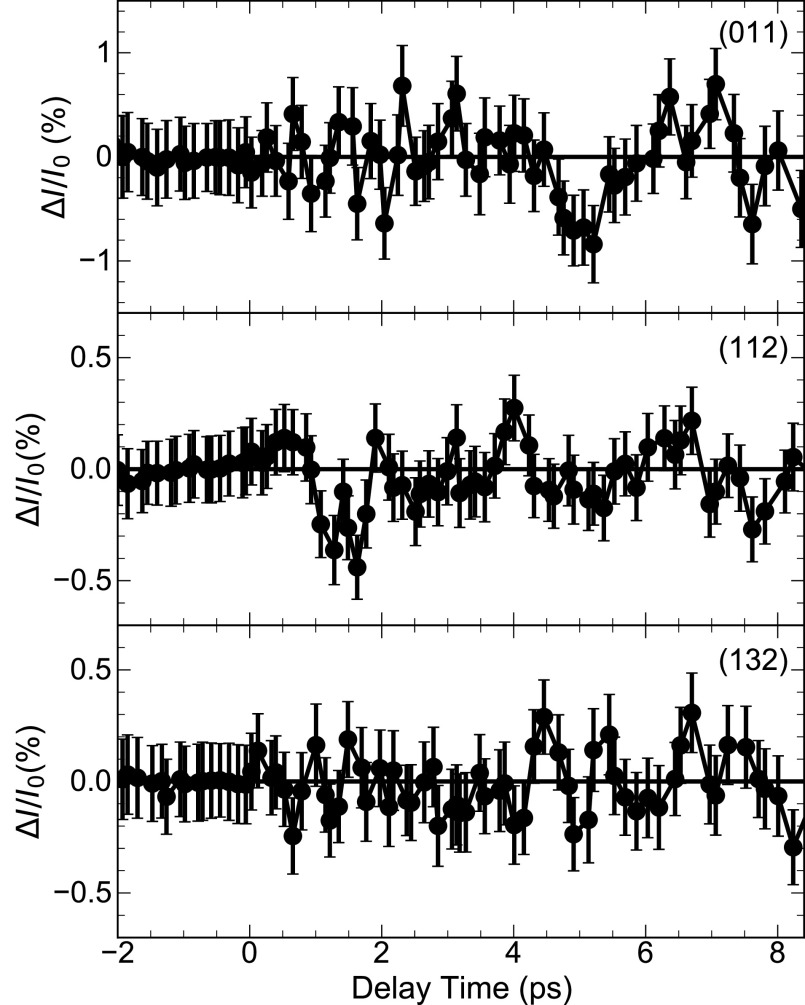
Transient intensity changes observed on the indicated powder diffraction peaks from KDP at *T *=* *295 K binned over a time interval of 200 fs (symbols).

**FIG. 4.**
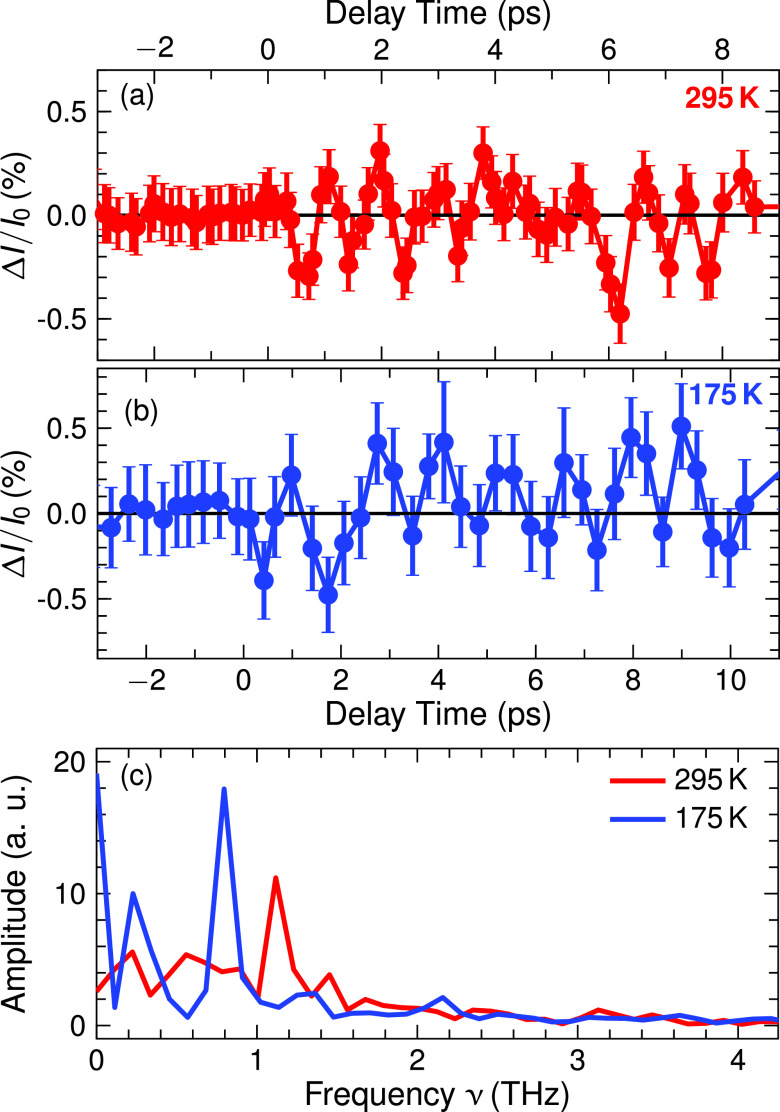
Transient intensity changes of the (020) diffraction peak at (a) 295 K and (b) 175 K. The data points represent values binned over a time interval of 200 fs in (a) and 400 fs in (b) around their delay position. (c) FFT of the transient intensity at 295 K with a peak frequency at 1.1 THz (295 K, in red) and at 0.78 THz (175 K, in blue).

In the pump-probe experiments, the 800-nm pump pulse excites phonons in the electronic ground state via impulsive Raman scattering. Upon excitation, the x-ray intensity on the different diffraction peaks changes, while their angular positions are preserved within the experimental angle resolution. In Figs. 2–4, x-ray intensity changes $\Delta I/I_{0}=(I-I_{0})/I_{0}$ on different diffraction peaks are plotted as a function of pump-probe delay (*I*, *I*_0_: intensity diffracted with and without excitation of the sample). Data points were recorded at 5754 and 1463 randomly chosen pump-probe delays for lattice temperatures *T *=* *295 K and 175 K, respectively. The maximum values of $\Delta I/I_{0}$ are on the order of 1%, while the smallest detectable value is approximately $10^{-3}$. This very high experimental sensitivity originates from a careful optimization of the experimental setup, and the long data recording times of up to 2.5 h per data point displayed in Figs. 2–4.

All transients display pronounced oscillatory intensity changes with a period close to 1 ps. Fourier spectra of the oscillatory components of the different transients are presented in panels (c) of Figs. 2 and 4. At *T *=* *295 K, one finds a main oscillation frequency of 1.1 ± 0.2 THz. A reduction of sample temperature from *T *=* *295 K to 175 K leads to a frequency shift from 1.1 to 0.78 THz, i.e., to a softening of the underlying phonon mode. Most striking is the persistence of the oscillatory intensity changes over a period of 10 ps, pointing to an underdamped character of the lattice motions inducing the changes of diffracted intensity.

**FIG. 5.**
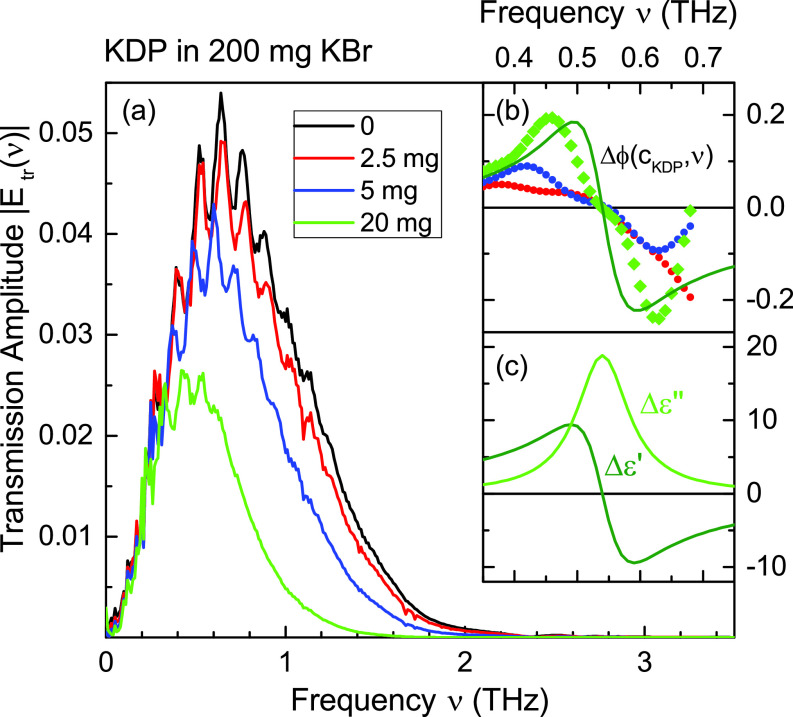
(a) THz-transmission amplitude $\left|E_{\text{tr}}(\nu )\right|$ at *T *=* *295 K of various pellets made of KBr and KDP crystallites with concentrations as indicated. (b) Colored symbols: reconstructed relative phase $\Delta \phi (c_{\text{KDP}},\nu )$ [cf. Eq.(A9)] for three different KDP concentrations. Green solid line: lorentzian fit to the relative phase measured with the highest KDP concentration (green symbols). (c) Real part Δε′ and imaginary part Δε″ of the dielectric function component originating from the resonance at 550 GHz.

Results from the THz experiment are summarized in Fig. 5. In panel (a), the amplitude of the transmitted THz field $\left|E_{\text{tr}}(\nu )\right|$ is plotted vs frequency *ν* for different concentrations $c_{\text{KDP}}$ [see Eq. (A1)] of KDP crystallites in the KBr pellet. There is a transmission decrease toward higher frequencies with increasing $c_{\text{KDP}}$, and all spectra are superimposed by Fabry–Pérot oscillations. The analysis of the spectra described in detail in Appendix A gives the frequency-dependent phase change Δϕ relative to the phase calculated with the dielectric function from Refs. 14 and 31 [panel (b)]. For all KDP concentrations, there is a zero crossing at 0.55 THz, pointing to an absorptive resonance in the imaginary part of the dielectric function of KDP. The solid green line in panel (b) is a Lorentzian fit to the measured phase changes, while the corresponding real part Δε′ and imaginary part Δε″ of the dielectric function are shown in panel (c).

### B. Transient charge density maps

**FIG. 6.**
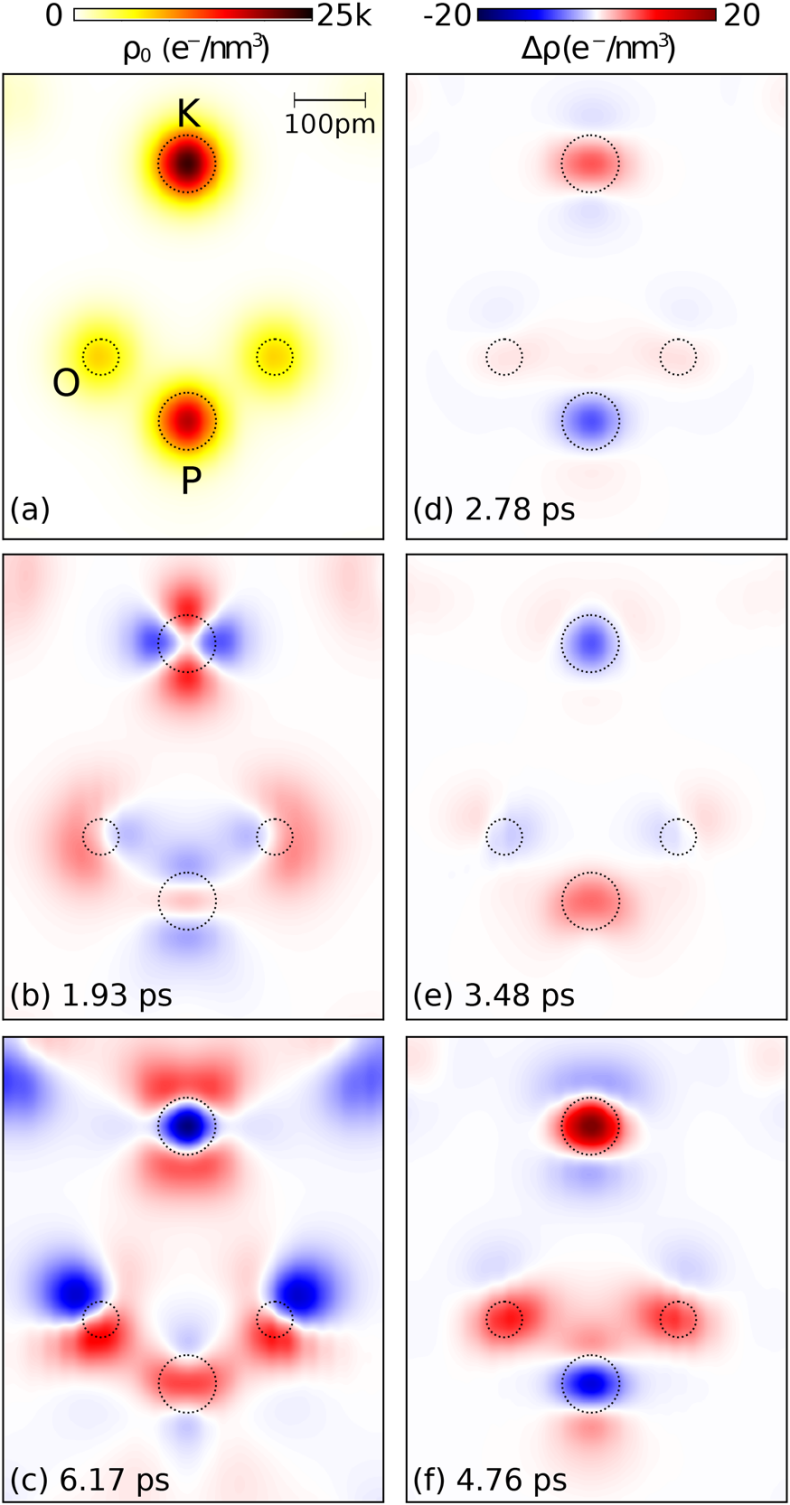
(a) Equilibrium electron density map at room temperature. $\rho_{0}(r)$ is shown in the gray plane of Fig. 1(a) containing the K, P, and the two upper O atoms from the phosphate group. (b)–(f) Transient electron density maps Δρ(r,t) at selected delay times *t* after impulsive-Raman excitation of the soft mode.

Transient electron density maps were derived from the x-ray diffraction data recorded at *T *=* *295 K, employing the maximum entropy method (MEM). This method provides differential density maps $\Delta \rho (r,t)=\rho (r,t)-\rho_{0}(r)$, where ρ(r,t) is the electron density at delay time t and $\rho_{0}(r)$ is the stationary electron density in the electronic ground state of PE KDP, serving as the so-called prior in the MEM. The structure factors and the atomic form factors related to the stationary $\rho_{0}(r)$ were calculated from the atomic positions reported in Ref. 27, taking into account the thermal motion of atoms at *T *=* *295 K. To adapt to the spatial resolution of the femtosecond experiment, which is limited by the maximum diffraction angle $2\theta_{\mathrm{hkl}}=58{}^{\circ}$, we multiply the structure factors of the high-resolution prior with a Gaussian weighting function, as described in detail in Ref. 32. Figure 6(a) displays the stationary electron density map *ρ*_0_(**r**) at *T *=* *295 K calculated in the plane indicated in gray in Fig. 1(a). This plane crosses the center of the unit cell along the *c* crystallographic axis containing a potassium, a phosphorous, and two oxygen atoms belonging to the central phosphate group. The electronic charge density is mainly concentrated on the atomic sites, but there is a small nonzero charge density in the interstitial region of the unit cell.

**FIG. 7.**
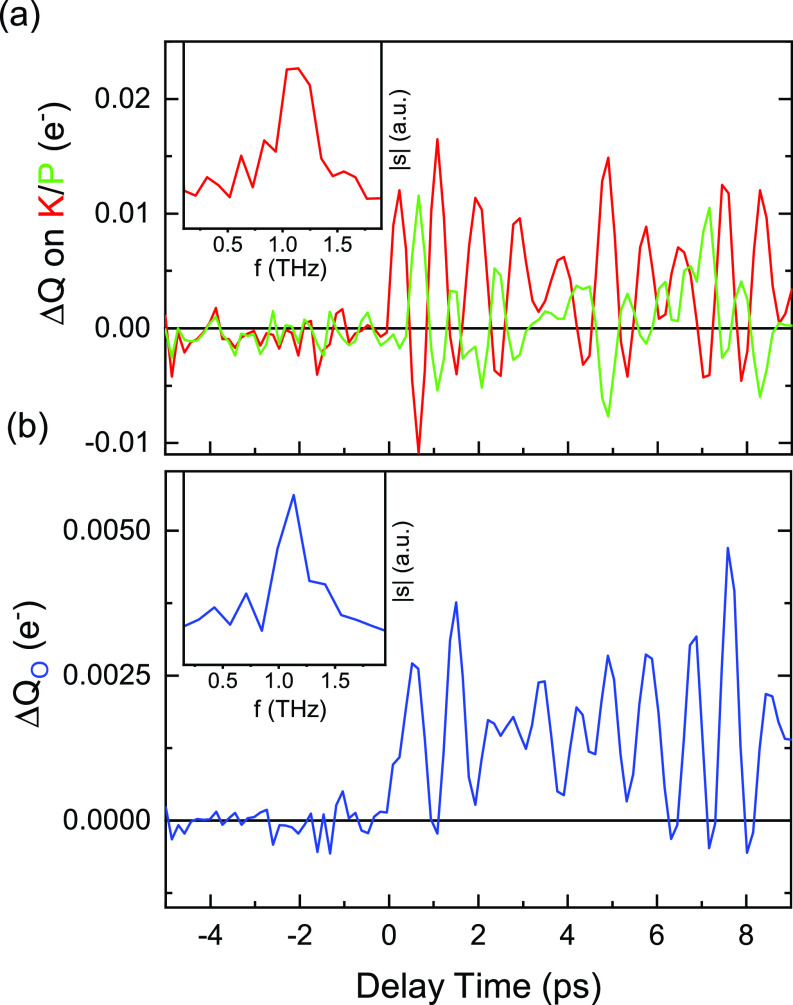
(a) Time evolution of the electronic charge change ΔQ integrated over the volume of the K (red line) and P atoms (green line). The charge changes show oscillations of opposite phase with a frequency of 1.1 THz. Inset: Fourier spectrum of the time-resolved ΔQ transient. (b) Same for an O atom of the $\text{P}{\text{O}}_{4}^{3-}$ unit.

For deriving Δρ(r,t) by the MEM, a three-dimensional grid was introduced, dividing the KDP unit cell into 72 × 72 × 72 voxels. Figures 6(b)–6(f) show transient electron density maps Δρ(r,t) for different time delays *t* in the same plane. The equilibrium spatial positions of each atom in the plane are shown by dashed circles. The transient charge-density maps [panels (b) to (f)] exhibit the following signatures:

(i) *Nuclear motions of the K*^+^ *ion along the c axis* lead to an elongation of charge density along the *c* axis, which is most clearly visible in panels (b) and (c). In the transient charge-density maps, the potassium motion shows up as a superposition of images of unit cells with K^+^ displacements in opposite directions. This superposition originates from the random relative orientation of the optical polarization of the pump pulses and the crystal axes of the individual crystallites.
(ii) *The occurrence of charge transfer between the potassium and phosphate ions* is clearly visible in panels (d)–(f) of Fig. 6. Electronic charge is shifted over the interatomic distance of several hundred picometers, while the atomic displacements connected with impulsive phonon excitations are in the sub-picometer range. The charge transfer shows an oscillatory character, in line with the periodic changes of diffracted intensity presented in Figs. 2 and 4. To bring out this behavior most clearly, we integrated the change of charge density over the different atomic volumes, giving the time-dependent changes of charge at the different sites (Fig. 7). Panel (a) shows the antiphasic oscillations of charge on the potassium and phosphorus sites with a frequency of 1.1 THz. The charge oscillations persist for a time range of at least 10 ps, due to the underdamped character of the related nuclear motions.
(iii) *An oscillatory charge transfer between the phosphorus and the oxygen atoms* occurs within the phosphate ion, as shown for the two in-plane oxygens in panels (d)–(f) of Fig. 6. The integrated charge change on one of such atoms plotted in Fig. 7(b) displays the same oscillation frequency of 1.1 THz as the other charge oscillations.
(iv) *At the sample temperature T =* 175 K, we observe very similar transient charge density maps (not shown) but with an oscillation frequency reduced to 0.78 THz. In other words, there is a softening of the underlying phonon mode with decreasing temperature.

Panels (b)–(f) of Fig. 6 correspond to different phases of the coherent nuclear and charge motions. Because of the random orientation of the crystallites in the powder sample, the spatially averaged diffraction pattern is not sensitive to the particular direction of charge transfer. As a result, the modulation frequency of diffracted intensity and charge density of 1.1 THz corresponds to the second harmonic of the underlying phonon frequency which, thus, has a value of 0.55 THz at *T *=* *295 K. It is important to note that the THz spectra presented in Fig. 5 exhibit a resonance at exactly this frequency.

## IV. DISCUSSION

The results of the femtosecond x-ray experiments demonstrate that impulsive phonon excitation of KDP crystallites in their electronic ground state induces pronounced relocations of electronic charge between the K^+^ and $\text{P}{\text{O}}_{4}^{3-}$ ions and between the P and O atoms within the $\text{P}{\text{O}}_{4}^{3-}$ groups. Such relocations occur over interatomic distances, which are orders of magnitude larger than the nuclear displacements connected with the excitation of phonons. This behavior is a clear hallmark of a soft-mode character of the underlying phonon, which manifests in concerted nuclear and charge motions, i.e., a mixed vibrational-electronic character. The strong elongations of electronic charge result in a minimization of electrostatic energy during atomic motion. Connected is a change of the macroscopic electric polarization to which the charge motions along the *c* axis make the main contribution.^33^

**FIG. 8.**
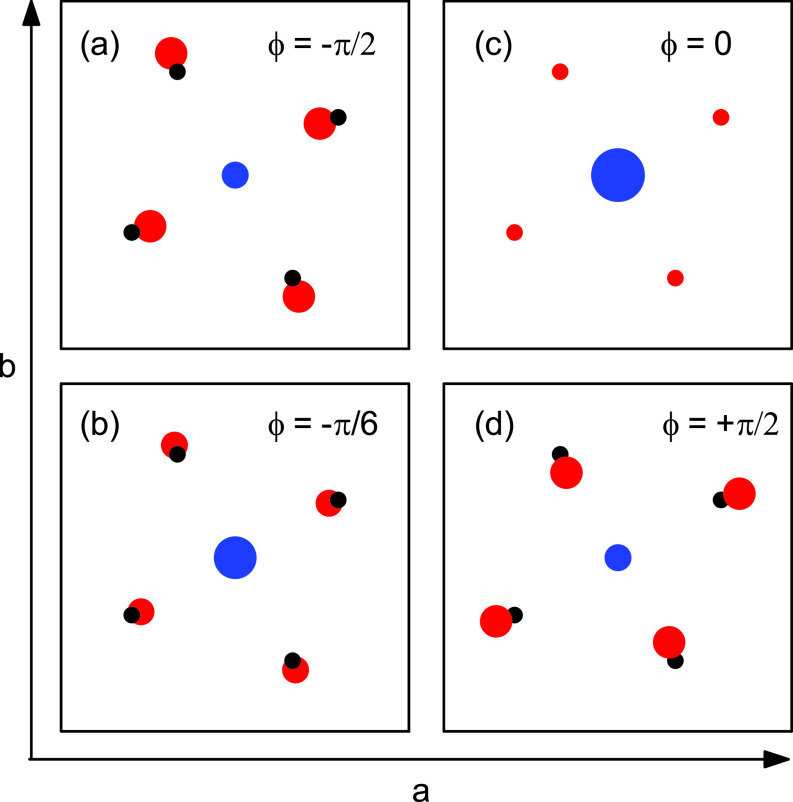
Cartoon of the soft mode motion with strongly exaggerated diplacements in the projection of the transient charge density maps onto the *ab* plane (black and red: oxygen atoms before and after excitation, blue: phosphorus atom). The size of the circles represents the charge density around the atomic position. The motions modulate the intensity of the (020) Bragg reflection shown in Fig. 4. It is a correlated motion of the oxygen atoms with *B*_2_ symmetry and a charge transfer from the phosphorus to the oxygens. Since the charge transfer happens for oscillation phases ϕ=−π/2 (a) and ϕ=+π/2 (d), the modulation of the (020) reflection occurs at the second harmonic of the soft-mode frequency $2\nu_{\text{sm}}=1.1$ THz.

The charge shift between the P and O atoms in the $\text{P}{\text{O}}_{4}^{3-}$ tetrahedra is strongly correlated with oxygen displacements of *B*_2_ symmetry in the *ab* plane. Such motions are part of the coherent nuclear elongations of the soft mode. In Fig. 8, we present a schematic view of the oxygen displacements by projecting the transient charge density maps onto the *ab* plane. Charge motions in this plane give rise to the intensity modulation on the (020) Bragg reflection [Fig. 4], which is insensitive to motions along the *c* axis. Panels (a) to (d) in which the atomic displacements are strongly enhanced show different phases of the soft-mode oscillation. For fixed charges on the P and O atoms, i.e., without interatomic charge transfer, the modulation amplitude on the (020) reflection would be less than 1% of the experimentally observed value. Thus, the charge transfer within the $\text{P}{\text{O}}_{4}^{3-}$ groups is an essential aspect of the soft-mode excitation and required to account for the observed x-ray intensity modulation.

The femtosecond pump pulses excite all Raman-active phonons within their bandwidth impulsively. In PE KDP, lattice modes belonging to the irreducible representations *A*_1_, *B*_2_, *B*_1_, and *E* of space group $I\bar{4}2d$ are Raman active.^33^ We do not observe in our data the appearance of forbidden reflections within our experimental sensitivity, as defined by an intensity of $0.15\cdot I_{\text{diamond}}$ with the intensity $I_{\text{diamond}}$ that of the reflection at 2θ=44° [Fig. 1(b)]. The absence of symmetry-forbidden reflections in the transient x-ray diffraction patterns shows that phonon excitations of *B*_1_ or *E* symmetry play a minor role. Of the remaining *A*_1_ and *B*_2_ symmetries, only *B*_2_ accounts for translational low-frequency motions of K^+^ and $\text{P}{\text{O}}_{4}^{3-}$ ions and related charge relocations along the *c* axis. We, thus, conclude that the prominent oscillations of diffracted x-ray intensity and oscillatory charge relocations are due to coherent nuclear motions along a *B*_2_ low-frequency phonon coordinate. The phonon frequency shifts from 0.55 THz at *T *=* *295 K to 0.39 THz at *T *=* *175 K, reflecting a mode softening with decreasing temperature in the PE phase.

The analysis of stationary Raman and infrared spectra of PE KDP has given a range of *B*_2_ phonon frequencies below 1 THz, which depend on the model applied for data analysis and are not fully consistent with each other. We recall that the THz spectra of KDP crystallites summarized in Fig. 5 suggest an absorption resonance at 0.55 THz, which we assign to a low-frequency optical phonon and which is in good agreement with the oscillation frequency found in the *T *=* *295 K x-ray transients. Femtosecond impulsive stimulated Raman scattering from bulk KDP has identified a transverse *B*_2_ phonon-polariton mode with a frequency of some 0.6 THz at *T *=* *270 K and approximately 0.4 THz at *T *=* *175 K.^22^ The phonon frequency decreases to less than 0.15 THz at $T-T_{C}=10$ K, in line with a soft-mode behavior. The frequencies reported in Ref. 22 agree well with the phonon frequencies observed in our x-ray experiments, which provide direct evidence for the soft-mode character via the transient charge density maps.

In *k* space, the coupling of light to LO and TO phonons leads to a polaritonic dispersion of the coupled quasi-particles. The photon dispersion $\nu =(c_{p}/2\pi )k$ of 800-nm light (*c_p_*: phase velocity in the crystal) and the (constant) TO phonon dispersion for a phonon frequency of ν=0.55 THz cross at $q_{c}\approx 173$ cm^−1^. For $q>q_{c}$, the lower polariton branch has a TO character. The impulsive stimulated Raman experiments of Ref. 22 were performed with a KDP single crystal and *q *=* *800 cm^−1^>*q*_c_ and, thus, also map the transient TO phonon excitation.

This picture changes for small crystallites of dimension *r* in the limit $r\cdot q_{c}\ll 1$. For r≈1 *μ*m as in our KDP powder sample, one estimates $r\cdot q_{c}\approx 0.0173\ll 1$. Under such conditions, the lowest polar phonon mode is connected with a spatially homogeneous polarization distribution in the crystallite, corresponding to an LO phonon excitation at *q* close to zero.^34–36^ In other words, the long-lasting charge oscillations found in the present experiments are due to impulsive excitation of a *longitudinal* Raman-active soft mode, which is observed here for the first time.

The ratio of the LO and TO phonon frequencies can be estimated with the help of the generalized Lyddane-Sachs-Teller relation^37^ and has a value of $\nu_{LO}=1.05\cdot \nu_{TO}=0.578$ THz. The small LO-TO frequency separation is within the spectral width of the THz TO resonance in Fig. 5(c) and of the Fourier transforms in Fig. 4(c). Thus, the LO frequency $\nu_{LO}=0.55$ THz derived from our experiments is very similar to the TO frequency found in Ref. 22.

We now address the damping behavior of the coherent soft-mode excitations. The oscillations of x-ray intensities diffracted from from the KDP crystallites KDP crystallites (Figs. 2 and 4) persist with minor changes of the maximum amplitudes over a 10-ps delay range, pointing to a damping time well beyond 20 ps. Such strongly underdamped soft-mode dynamics is observed here for the first time and in sharp contrast to the established picture of overdamped soft modes in KDP. It should be noted that the transverse *B*_2_ phonon of bulk KDP studied in Ref. 22 shows a substantially shorter damping time of approximately 1 ps over a wide range of sample temperatures.

There are different mechanisms that contribute to damping of the oscillatory intensity changes, which represent a macroscopic average over all crystallites excited in the powder. First, there may be a spread of phonon frequencies in crystallites of different sizes and shapes, i.e., an inhomogeneous broadening, which results in a loss of mutual phase between contributions from different crystallites. Second, damping of the coherent phonon oscillations *per se* is connected with a decay of quantum coherence between soft-mode excited states. For TO modes, both pure dephasing and population relaxation via anharmonic and electric coupling to other phonons contribute. The ionic character of KDP and, in particular, the hybrid nuclear-electronic nature of soft modes make fluctuations of the local electric fields in the crystal a predominant source of decoherence of TO modes. Such fluctuations originate from stochastic nuclear motions along a multitude of thermally activated phonon coordinates and from scattering processes with electrons, typically resulting in a faster dephasing of TO than LO modes.

In contrast to TO coherences, decoherence of LO excitations requires an energy exchange in the interaction with other excitations, e.g., during energy and population relaxation. At the low LO frequency of 0.55 THz observed here, the decay into acoustic phonons via anharmonic coupling is expected to represent the main channel of energy relaxation. Extrapolating from the acoustic phonon dispersion of FE KDP presented in Ref. 38, such *k*-conserving decay processes occur in a *k* space volume of less than 1% of the first Brillouin zone with a comparably small number of acoustic phonon states. As a result, one expects relaxation times beyond 1 ns for this decay mechanism.

This qualitative discussion underlines that a frequency- and *q*-dependent dielectric function is required to properly account for the decoherence and population relaxation of TO and LO soft modes. Work along such lines has remained very limited, and a quantitative analysis requires much more detailed experimental information on the transient dielectric response of KDP.

## V. CONCLUSIONS

The results presented here reveal a highly underdamped longitudinal soft mode in paraelectric KDP crystallites. The soft-mode character of this phonon with a frequency of 0.55 THz at *T *=* *295 K and 0.39 THz at *T *=* *175 K is evident from transient charge density maps, which are derived from femtosecond x-ray powder diffraction data. Charge relocation occurs over interatomic distances of hundreds of picometers, while nuclear displacements are in the sub-picometer regime. An oscillatory charge transfer between the K^+^ and $\text{P}{\text{O}}_{4}^{3-}$ ions along the *c* axis of the unit cell is accompanied by charge transfer between the phosphorus and oxygen atoms in the $\text{P}{\text{O}}_{4}^{3-}$ units, both directly modulating the macroscopic electric polarization. In the crystallites, the soft mode has a longitudinal character and displays a much weaker damping of coherent oscillations than its transverse counterpart in bulk KDP. This behavior is a manifestation of the wavevector dependence of the dielectric function of KDP, an issue that needs further experimental and theoretical investigation.

## Figures and Tables

**FIG. 9.**
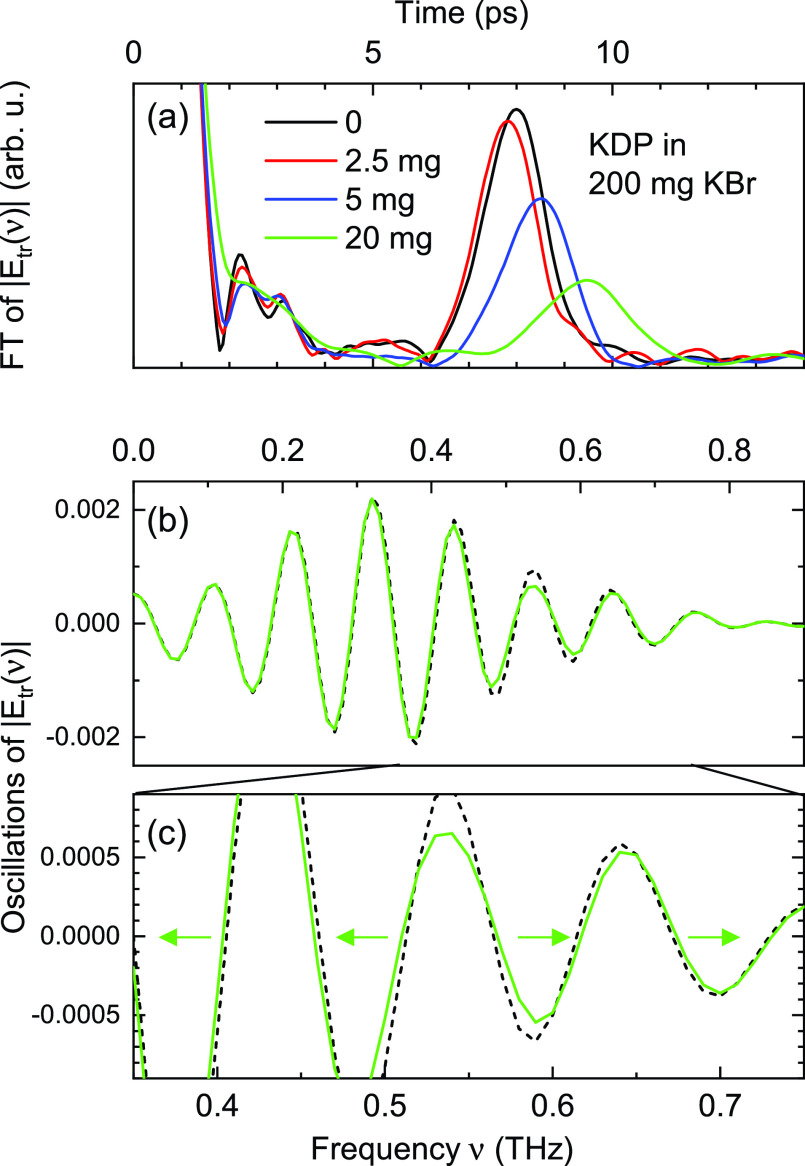
(a) Fourier transforms of the THz-transmission amplitude $\left|E_{\text{tr}}(\nu )\right|$ shown in Fig. 5(a). (b) Green solid line: oscillatory contribution of the green curve in Fig. 5(a). Black dashed line: expected oscillations according to (A9) for the 20-mg pellet calculated from the transmission of the pure KBr pellet and dielectric functions from Refs. 14 and 31. (c) Zoom of the frequency range at which the amplitude change and phase shifts between data and expected oscillations are more obvious. The green arrows indicate the expected phase shifts below the resonance (i.e., larger refractive index of the pellet) and above the resonance (i.e., smaller refractive index).

**FIG. 10.**
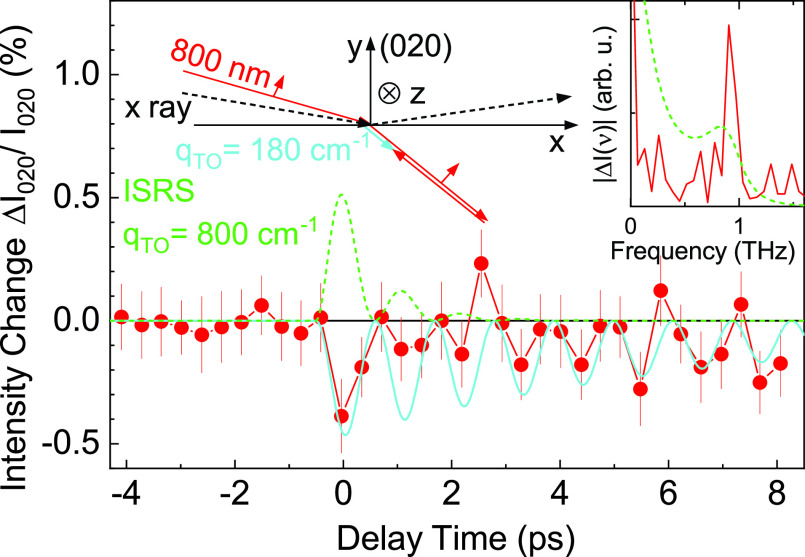
Femtosecond x-ray diffraction on a bulk KDP single crystal at *T *=* *300 K (symbols) with a schematic of the experimental geometry. The transient change of intensity $\Delta I_{020}(t)/I_{020}$ of the (020) Bragg reflection is plotted as a function of pump-probe delay between p-polarized 800-nm pump and femtosecond x-ray probe pulses. Inset: FFT of the x-ray experimental data (red) and from the ISRS experiments in Ref. 22 (green). Simultaneous presence of *x* and *y* contributions to the 800-nm driving field allows for ISRS of the transverse *B*_2_ soft mode with a wave vector of $q_{\text{TO}}\approx 180$ cm^−1^ in the KDP bulk crystal. Blue curve: fit with slightly damped oscillations with $\nu_{\text{osc}}\approx 0.9$ THz. Green curve: strongly damped transverse *B*_2_ soft mode oscillations with a wave vector of $q_{\text{TO}}\approx 800$ cm^−1^ as obtained in ISRS experiments of Ref. 22.

## Data Availability

The data that support the findings of this study are available from the corresponding author upon reasonable request.

## APPENDIX A: LINEAR TERAHERTZ RESPONSE OF THE *B*_2_ SOFT MODE OF KDP CRYSTALLITES IN KBr

To detect a sharp absorptive resonance superimposed on a broad absorptive background and to exclude potential artifacts from the absorption of residual water vapor in the setup, we analyzed exclusively the spectral phase of the Fabry–Pérot oscillations due to the round-trips within the KDP/KBr pellets. Performing a Fourier transform of the THz amplitude spectra presented in Fig. 5 results in the transients shown in Fig. 9(a). We extracted the respective first replica occurring around 8 to 9 ps by an appropriate Gaussian filter in the time domain and performed a back transform to the spectral domain, which is exemplarily shown for the green curve in panels (b) and (c). The green arrows in (c) indicate the expected phase shifts for a narrow absorptive resonance, i.e., a larger refractive index below and a smaller refractive index above the resonance. The quantitative analysis was done as follows: the peak positions and amplitudes of the Fabry–Pérot oscillations depend on the thickness *L* of the pellet and the frequency-dependent complex refractive index of the respective mixture with the volume fraction,

$$c_{\text{KDP}}=\frac{V_{\text{KDP}}}{V_{\text{KDP}}+V_{\text{KBr}}}=\frac{m_{\text{KDP}}/\rho_{\text{KDP}}}{m_{\text{KDP}}/\rho_{\text{KDP}}+m_{\text{KBr}}/\rho_{\text{KBr}}}.$$
(A1)

The refractive index of the mixture $n_{\text{mix}}(c_{\text{KDP}},\nu )=\sqrt{\varepsilon_{\text{mix}}(c_{\text{KDP}},\nu )}$ determines both the single-pass transmission amplitude

$$t_{\text{sp}}(c_{\text{KDP}},\nu )=\text{exp}\;\left[i\frac{2\pi \nu L}{c}n_{\text{mix}}(c_{\text{KDP}},\nu )\right]$$
(A2)

and the reflectivity of the pellet–air interface, which can be approximated by the pure KBr reflectivity:

$$r_{\text{mix}}(c_{\text{KDP}},\nu )\approx r(\nu )=\frac{n_{\text{mix}}(0,\nu )-1}{n_{\text{mix}}(0,\nu )+1}.$$
(A3)

The transmitted electric field including the Fabry–Pérot oscillation reads

$$E_{\text{tr}}(c_{\text{KDP}},\nu )=\frac{E_{\text{in}}(\nu )\;t_{\text{sp}}(\nu )\left[1-r^{2}(\nu )\right]}{1-r^{2}(\nu )\;t_{\text{sp}}^{2}(\nu )},$$
(A4)

$$\begin{array}{c} \approx \;E_{\text{in}}(\nu )\;t_{\text{sp}}(\nu )\left[1-r^{2}(\nu )\right]\\ \times \left[1+r^{2}(\nu )t_{\text{sp}}^{2}(\nu )+r^{4}(\nu )t_{\text{sp}}^{4}(\nu )+\cdots \right] \end{array},$$
(A5)

$$E_{\text{fp}}(c_{\text{KDP}},\nu )\approx -2\;|E_{\text{in}}(\nu )\;t_{\text{sp}}^{3}(\nu )r^{2}(\nu )|\;\text{cos}\;\left(\text{arg}\left[t_{\text{sp}}^{2}(\nu )\right]\right),$$
(A6)

$$\begin{array}{c} \varepsilon_{\text{mix}}(c_{\text{KDP}},\nu )=\varepsilon_{\text{KBr}}(\nu )+3\;c_{\text{KDP}}\;\varepsilon_{\text{KBr}}(\nu )\frac{\left\langle \varepsilon_{\text{KDP}}(\nu )\rangle -\varepsilon_{\text{KBr}}(\nu \right)}{\left\langle \varepsilon_{\text{KDP}}(\nu )\rangle +2\;\varepsilon_{\text{KBr}}(\nu \right)} \end{array},$$
(A7)

$$\langle \varepsilon_{\text{KDP}}(\nu )\rangle =\frac{\varepsilon_{\text{KDP}}^{c}(\nu )+2\;\varepsilon_{\text{KDP}}^{a}(\nu )}{3}.$$
(A8)

In the Taylor expansion (A5) of the electric field transmitted through the pellet (A4), we take the fundamental term $r^{2}(\nu )t_{\text{sp}}^{2}(\nu )$ of the Fabry–Pérot oscillation (A6), whose phase is encoded in the cosine function containing the wanted information on the real part of the refractive index $\text{Re}\left[n_{\text{mix}}(c_{\text{KDP}},\nu )\right]$ in (A2). The oscillation amplitude in front of the cosine contains the incident THz field $E_{\text{in}}(\nu )$ and the reflectivity r(ν). The amplitude of the single pass propagation within the pellet $\left|t_{\text{sp}}(\nu )\right|$ [Eq. (A2)], which accounts for the absorption in the pellet, does not play any role in the phase analysis of the Fabry–Pérot oscillations.

The main advantage of analyzing the resonance through the spectral phase of the Fabry–Pérot oscillations is that it avoids any artifacts stemming from water-vapor absorption lines occurring in the same spectral range.

Equation (A7) [i.e., Eq. (9.7) of Ref. 39] describes the dielectric function of an emulsion of spherical KDP crystallites in a KBr environment, an approach that is valid for small concentrations $c_{\text{KDP}}$.

For randomly oriented KDP crystallites in the pellets, the orientationally averaged dielectric function (A8) determines via (A7) the complex refractive index $n_{\text{mix}}(c_{\text{KDP}},\nu )$ in (A2).

Next, using the dielectric functions from Refs. 14 and 31 in Eqs. (A7) and (A8), we multiply the oscillatory contributions observed for the pure KBr pellet with the expected result according to $c_{\text{KDP}}$ and Refs. 14 and 31,

$$\begin{array}{c} E_{\text{expected}}(c_{\text{KDP}},\nu )=E_{\text{tr}}(c_{\text{KDP}}=0,\nu )\;\text{exp}\;\left[i\frac{4\pi \nu L}{c}n_{\text{mix}}(c_{\text{KDP}},\nu )-i\frac{4\pi \nu L_{\text{KBr}}}{c}n_{\text{mix}}(0,\nu )\right] \end{array}$$
(A9)

and adjusted the thickness *L* of the KDP-containing sample to get the best fit with the experimentally observed Fabry–Pérot oscillations. We get an almost perfect agreement of both the phase and amplitude of the Fabry–Pérot oscillations except in a narrow spectral range around $\nu_{\text{sm}}\approx 0.55$ THz [cf. Figures 9(b) and 9(c)], which points to an additional narrow resonance at that frequency. This spectral range was not covered by the measurements presented in Ref. 14. In the experiments shown in Fig. 1(a) of Ref. 15 (i.e., the paraelectric phase), this resonance becomes only visible for lower temperatures.

The experimentally observed additional resonance can be extracted by calculating the phase difference $\Delta \phi (c_{\text{KDP}},\nu )=\text{arg}[E_{\text{tr}}(c_{\text{KDP}},\nu )/E_{\text{expected}}(c_{\text{KDP}},\nu )]$, which is shown for three different concentrations in Fig. 5(b) as colored symbols. Solving backwards Eqs. (A9), (A2), (A7), and (A8), one can reconstruct the additional contribution to the longitudinal dielectric function Δε(ν) along the *c* axis of KDP at *T *=* *300 K in the spectral range between 0.35 and 0.75 THz. The real (green solid line) and imaginary parts (green dashed line) of the best fit with a Lorentz oscillator are shown in Fig. 5(c).

## APPENDIX B: WHY IS THE CHARACTER OF THE SOFT MODE IN SMALL CRYSTALLITES PREDOMINANTLY LONGITUDINAL?

Optical phonons with an almost homogeneous lattice polarization in a small crystallite have a predominant longitudinal character. The theory for polariton modes of small spheres described in Sec. II of Ref. 36 is helpful to support this fact. In finite crystals, such optical phonons have always a mixed longitudinal-transverse character. To estimate the different contributions to such a quasiparticle, one can decompose the “electric mode” with *l *=* *1 [Eq. (28) in Ref. 36] into plane wave bulk LO and transverse polariton modes. The longitudinal contributions are responsible for the (small) space charge within the sphere and the accumulated charge on its surface occurring during phonon oscillation. The transverse contributions (being responsible for THz absorption) are strictly related to the amplitude of the oscillating magnetic field within the sphere and are very small in the case of small crystallites (1 *μ*m diameter) and phonon frequencies in the GHz range. The predominant longitudinal contributions to the quasiparticle result in (i) an LO-TO splitting [Eq. (55) in Ref. 36 with $\varepsilon_{M}=1,\;\varepsilon_{\infty }=21$, and $\varepsilon_{0}=22.5$ at room temperature] almost perfectly matching that of the bulk crystal, (ii) a negligible size dependence [Eq. (56) in Ref. 36] suppressing effectively inhomogeneous broadening in the powder sample, and (iii) allowing for an extremely weak damping of coherent oscillations, since elastic scattering processes between the contributing charged particles cannot contribute to the damping of longitudinal elementary excitations.

## APPENDIX C: TRANSVERSE OPTICAL *B*_2_ SOFT MODE OSCILLATIONS IN A SINGLE KDP BULK CRYSTAL

In addition to the femtosecond x-ray powder diffraction experiments discussed above, we performed time-resolved x-ray diffraction experiments on a single KDP bulk crystal at *T *=* *300 K. The results are shown as red symbols in Fig. 10 together with an inset explaining the experimental geometry. P-polarized 800-nm pump pulses enter the KDP crystal having both *x* and *y* contributions in their driving field within the crystal, which allows for impulsive stimulated Raman scattering (ISRS) in forward geometry, thereby exciting coherent transverse (TO) *B*_2_ soft mode oscillations with a very small wave vector of only $q_{\text{TO}}\approx 180$ cm^−1^.

The transient intensity change $\Delta I_{020}(t)/I_{020}$ of the (020) Bragg reflection (symbols) is plotted as a function of pump-probe delay between 800-nm pump and femtosecond x-ray probe pulses. For the correspondingly small Bragg angle, dynamical x-ray diffraction theory predicts (for perfect crystals) an extinction length of only 1.5 *μ*m for the (020) Bragg reflection, i.e., only a very thin layer of photo-excited KDP is probed in such an experimental geometry.

A fit (blue curve) with a frequency of $\nu_{\text{osc}}\approx 0.9$ THz and a damping time of 7.8 ps is in fair agreement with the weak damping of the excited transverse *B*_2_ soft mode oscillations at a wave vector of $q_{\text{TO}}\approx 180$ cm^−1^. This behavior is similar to their longitudinal counterpart experimentally observed in the transient powder diffraction experiments discussed above.

For comparison, we plot the green curve showing strongly damped transverse *B*_2_ soft mode oscillations with a wave vector of $q_{\text{TO}}\approx 800$ cm^−1^ as obtained in ISRS experiments of Ref. 22. Note that the soft mode in ISRS measurements shows a well quadratic dependence. Such a comparison shows that the wavelength of coherent transverse *B*_2_ soft mode oscillations in paraelectric KDP plays a crucial role concerning the damping of such oscillations. Long wavelengths correspond to large volumes with unidirectional electric currents connected to the soft-mode oscillation. In contrast, counterpropagating currents in alternating thin sheets, i.e., soft-mode excitations of short wavelength, are additionally exposed to elastic scattering processes, which can induce an efficient dephasing of coherent soft-mode excitations.
